# Malignant Phyllodes Tumor of the Breast in a 26-year-old Woman

**DOI:** 10.7759/cureus.6590

**Published:** 2020-01-07

**Authors:** Nga T Nguyen, Lynsey M Maciolek, Suimin Qiu, Sarfaraz Sadruddin, Quan D Nguyen

**Affiliations:** 1 Radiology, University of Texas Medical Branch, Galveston, USA; 2 Pathology, University of Texas Medical Branch, Galveston, USA

**Keywords:** malignant phyllodes tumor, myofibrosarcoma, fibroadenoma, phyllodes tumor, borderline phyllodes tumor, breast carcinoma, leaf-like projections, breast sarcoma, p53 gene, li-fraumeni syndrome

## Abstract

Breast cancer is the most commonly diagnosed cancer and the second leading cause of cancer death in women. Early detection, accurate diagnosis and proper treatment are important prognostic factors due to the wide variety of breast cancer subtypes. This becomes of particular importance with rare breast tumors, which are difficult to diagnose due to their varying presentations. Malignant phyllodes tumor (PT) is one of the rare breast tumors that is difficult to diagnose. First reported in 1838, PT of the breast accounts for less than 1% of all breast masses. Based on histological features, PT is categorized on a spectrum of benign to malignant tumors. High-grade PTs are commonly seen in older patients but can also occur in young patients, as reported in this case of a 26-year-old female. Failure to detect the malignancy early and initiate appropriate treatment can lead to widespread metastasis and poor outcomes.

## Introduction

In the past few decades, with easier access to high-quality prevention, early detection and advanced treatment, breast cancer mortality rates have declined significantly, dropping 40% from 1989 to 2017 [1]. While treatments for common types of breast cancer have been extensively studied and made available to patients, the pathogenesis and course of treatment for rarer breast tumors have not been well established. Phyllodes tumors (PTs) of the breast belong to one of those rare entities.

PTs of the breast account for 0.3% to 1% of all breast tumors [2]. They are most commonly diagnosed in the elderly population. Unlike the more common type of carcinoma caused by neoplastic epithelial cells, these tumors originate from the connective tissue, stroma, which provides the supportive framework for the lobules, ducts, blood vessels and lymph. PTs of the breast are classified by the World Health Organization into benign, borderline and malignant with varying presentations, prognoses and courses of treatment [3]. While malignant PTs can metastasize with poor outcomes and high rates of recurrence, benign and borderline tumors present with good prognosis and lower rates of recurrence [2]. The classification of PT is established based on specific histological criteria, including cellular atypia, number of mitoses, nuclear uniformity, stromal cellularity and border infiltration. Nevertheless, the differentiation between PT subtypes and the respective differential diagnoses is not without controversy.

Because of the challenges in providing an accurate diagnosis and the predilection of PT in the older population, malignant PTs in the younger generations can be overlooked. Different subtypes of PT have different courses of treatment; therefore, underdiagnosis or overdiagnosis can lead to inadequate or unnecessary interventions. Cases of malignant PTs in young patients have been reported, but it is very rare for a patient to be diagnosed with both borderline and malignant PTs. This paper details the presentation of malignant PT of the right breast in a very young woman with a past medical history of sarcoma and borderline PT of the left breast. Highlights of imaging features, pathohistological findings, basic histochemistry, differential diagnoses and management are also presented.

## Case presentation

**Figure 1:**
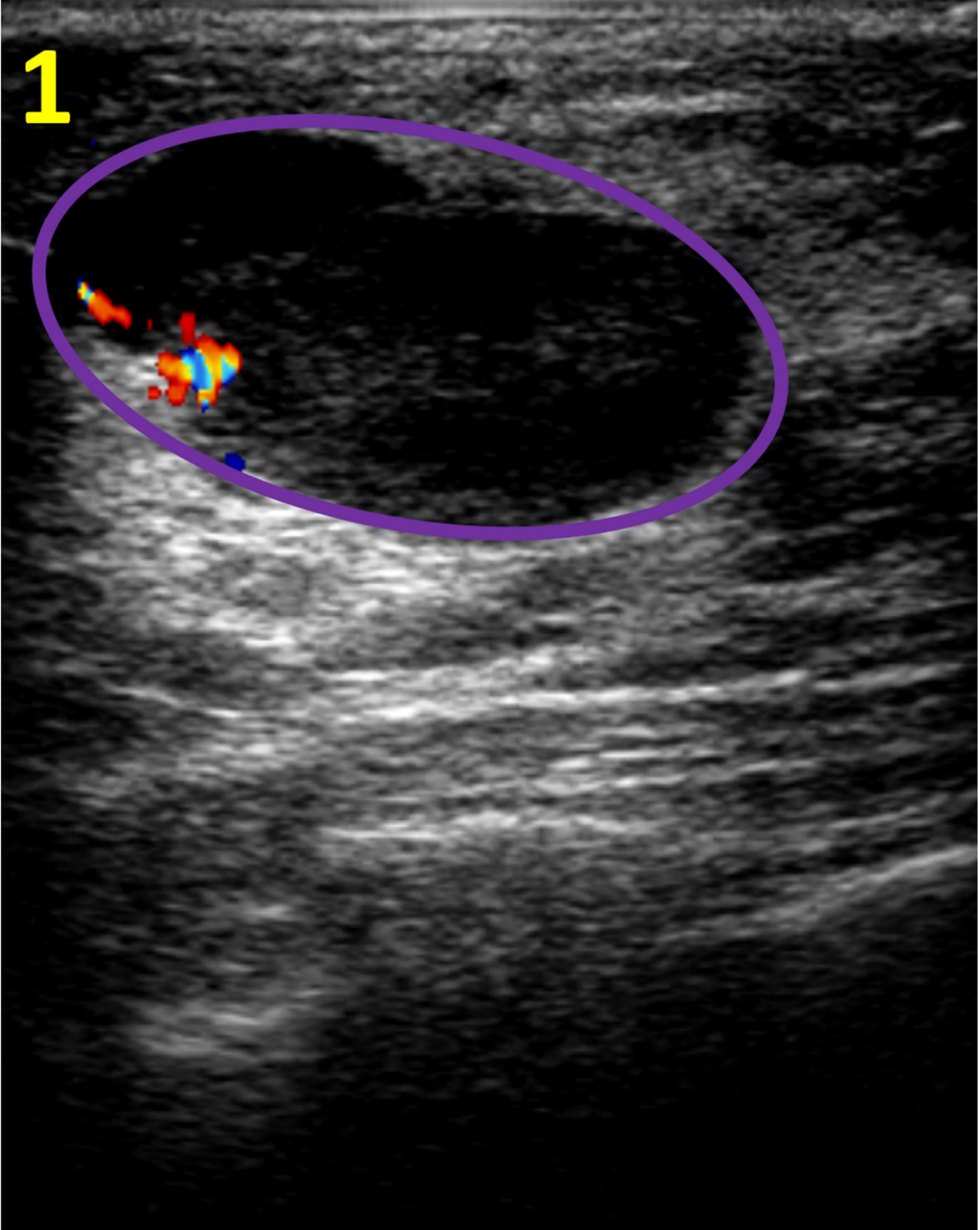
Targeted Ultrasound of the Right Breast Targeted ultrasound of the right breast demonstrates a solid mass (purple circle) measuring 26 x 20 x 16 mm at the site of the clinically palpable mass at three o'clock at a distance of 2 cm from the nipple. The characteristics of the finding include a hypoechoic pattern, an abrupt interface, circumscribed margins, an orientation that is parallel to the skin line, a combined pattern of posterior acoustics and an oval shape.

A 26-year-old woman presented with an enlarging tender mass in the right breast. Her past medical history was significant for multiple breast tumors. At age 19 years, a sarcoma of the left breast arising from a tubular adenoma was discovered. At that time, she underwent a segmental mastectomy. At age 20 years, she was diagnosed with a borderline PT of the same breast, treated with a nipple sparing mastectomy of the left breast. The patient’s family history was significant for breast cancer in two maternal aunts. On physical examination of the right breast, a moveable mass at three o’clock at a distance of 2 cm from the nipple was palpated. A targeted ultrasound of the right breast revealed a hypoechoic mass at the site of the clinically palpable mass with an abrupt interface, circumscribed margins, a combined pattern of posterior acoustics and an oval/lobulated shape, as seen in Figure 1.

**Figure 2:**
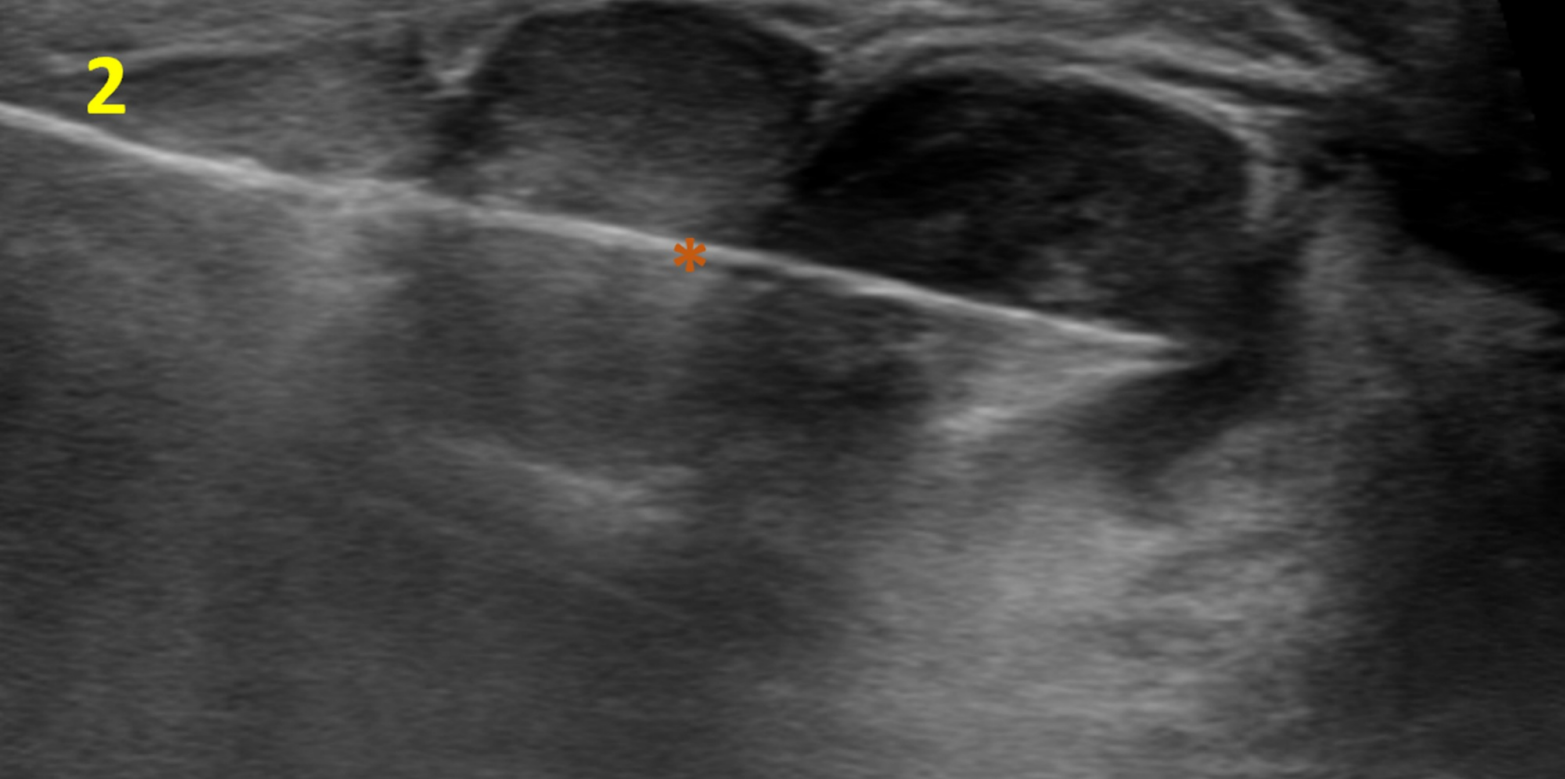
Ultrasound-guided Core Needle Biopsy of the Right Breast The hyperechoic biopsy needle (orange asterisk) is visualized within the right breast mass.

**Figure 3:**
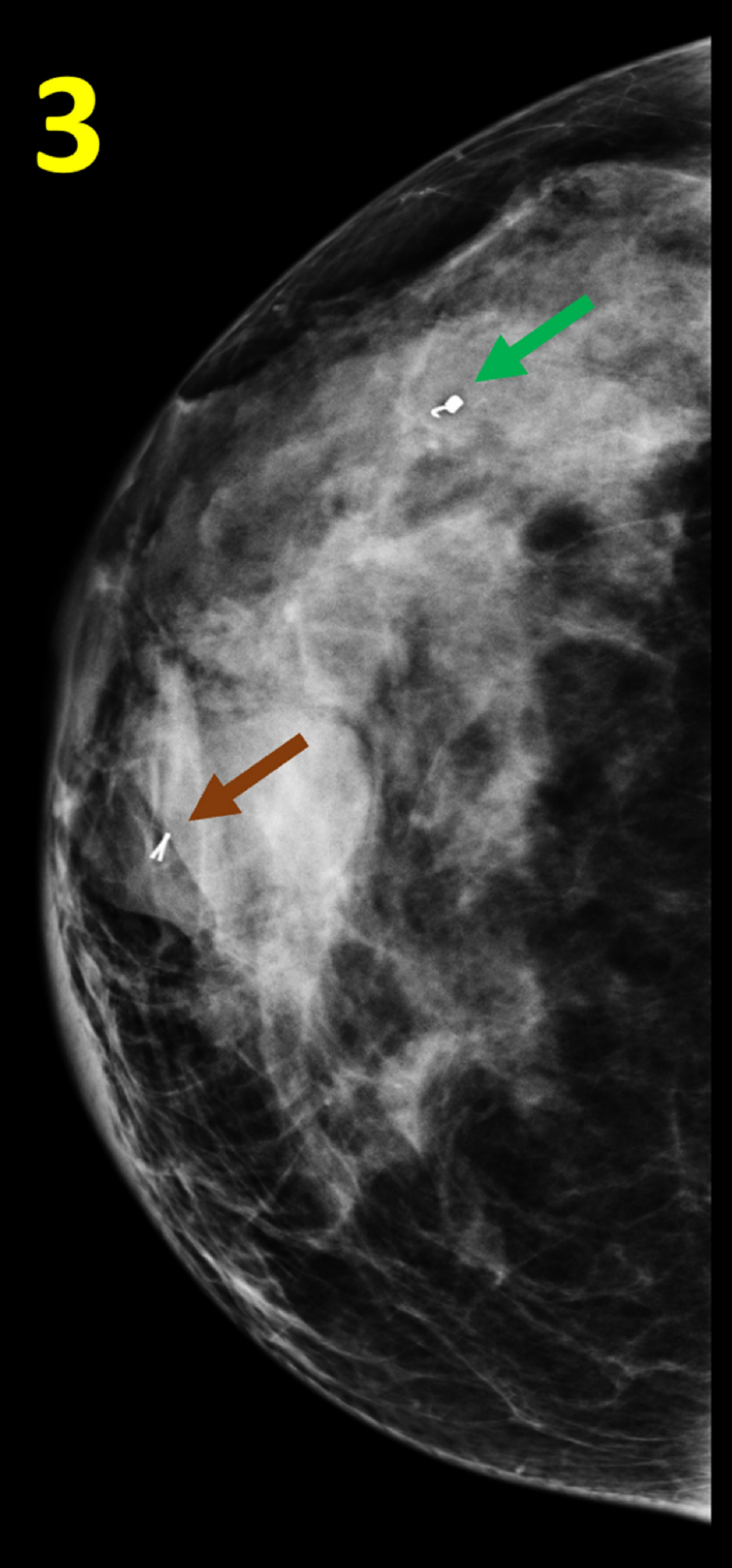
Post-biopsy Mammogram of the Right Breast in Cranial-caudal View A micromarker (RIBBON) was placed at the site of the core biopsy. The clip (RIBBON) (brown arrow) was present within the mass. An additional clip (COIL) (green arrow) was noted in the upper outer quadrant of the right breast. The patient could not provide any history regarding this biopsy.

The right axilla demonstrated no lymphadenopathy. Because of her significant past medical history, current presentation of an enlarging right breast mass and the sheer size of the tumor, primary breast malignancy was raised as a concern. Subsequently, an ultrasound-guided core needle biopsy of the right breast was performed for a more accurate tissue diagnosis (Figures 2, 3).

**Figure 4:**
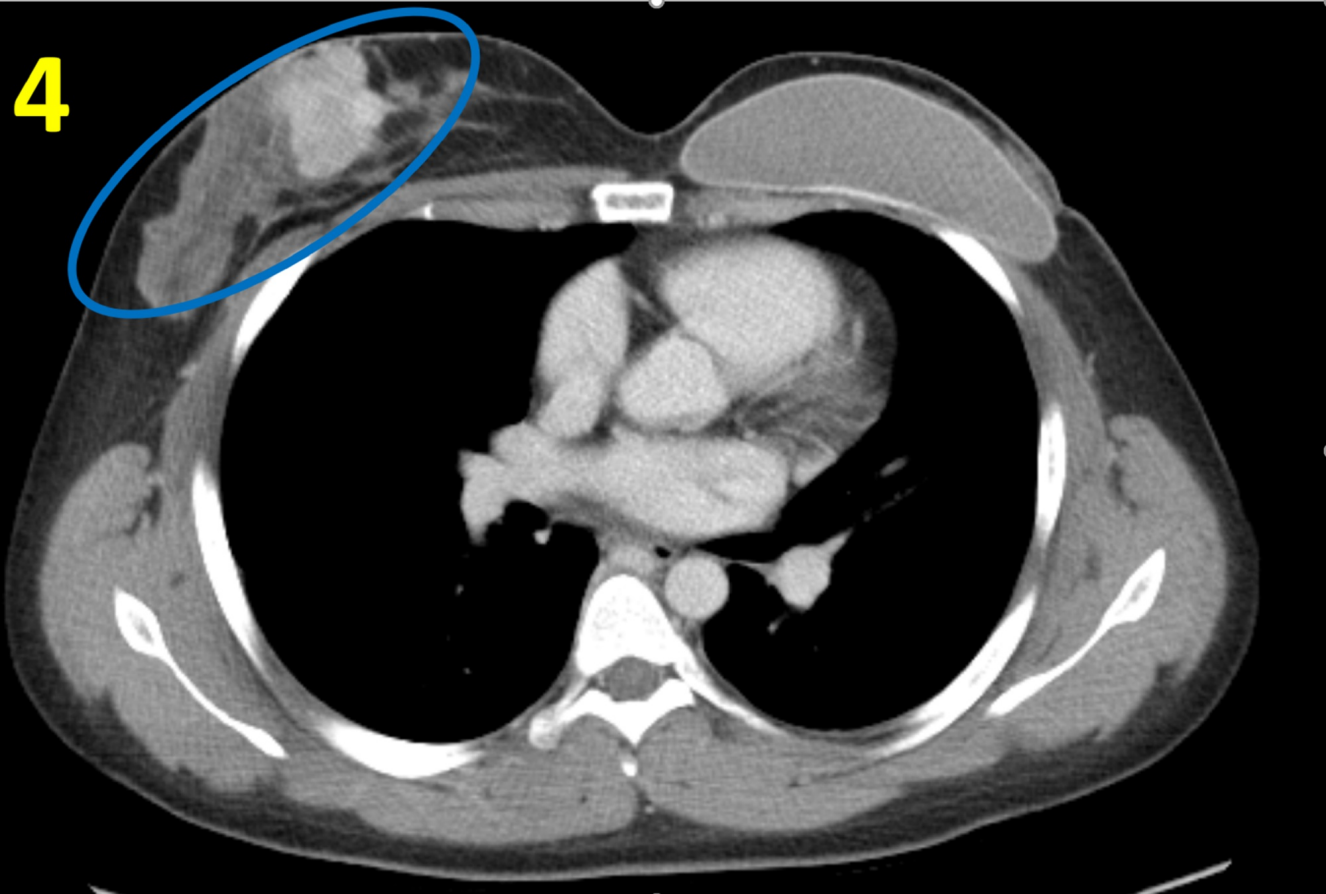
Computed Tomography Scan of the Thorax An enhancing mass (blue circle) was seen in the right breast, in concordance with the right breast malignancy. No evidence of metastatic disease was found.

Histology of the biopsy revealed sheets of basophilic spindle cells with high nuclear-to-cytoplasmic ratios, atypical mitoses and frequent prominent nucleoli. Malignant stromal cells were seen infiltrating between benign-appearing glans. Due to the high suspicion for malignancy, a computed tomography scan of the thorax, abdomen and pelvis was performed to assess for metastasis (Figure 4).

**Figure 5:**
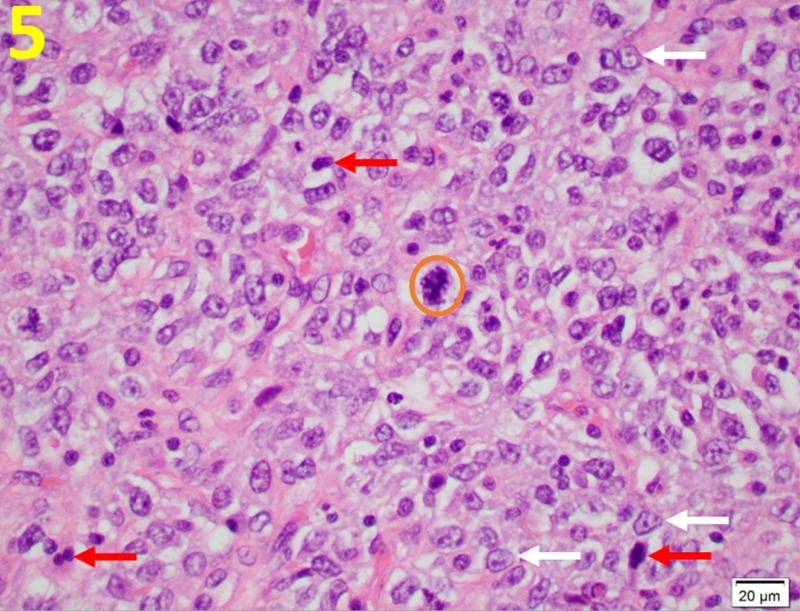
Nuclear Pleomorphism in Malignant Phyllodes Tumors The stroma cells show marked atypia with numerous mitoses (orange circle), prominent nucleoli (white arrows) and high nuclear-to-cytoplasmic ratio (red arrows). This is demonstrated on a hematoxylin and eosin stain at a magnification of 400x.

This scan indeed ruled out metastatic disease in this patient. The patient eventually had a simple mastectomy of the right breast. Histology of the mastectomy tissue demonstrated hypercellularity with malignant stromal cells and increased mitotic activity (30 mitoses per 10 high-power fields) (Figure 5).

**Figure 6:**
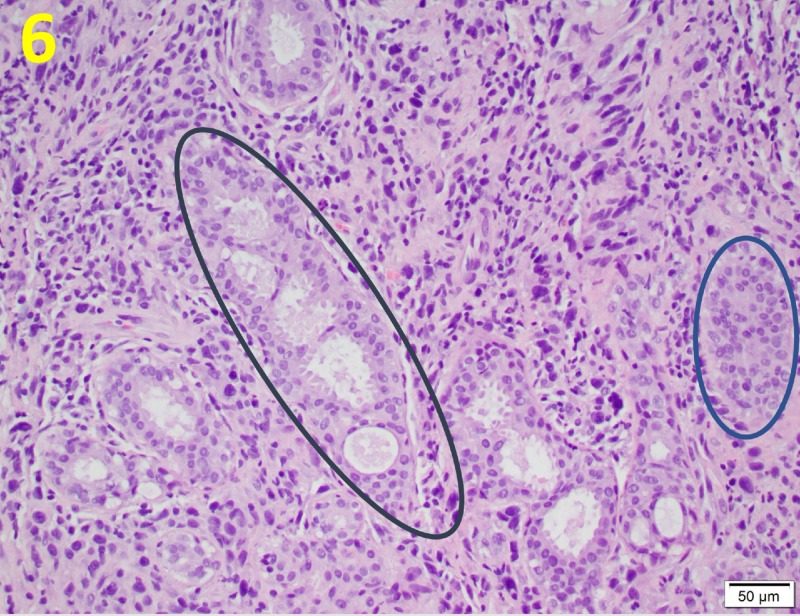
Malignant Stromal Cell Proliferation The presence of benign epithelium and periductal condensation of atypical stromal cells (black circle) is typical of malignant phyllodes tumors. There is also evidence of multinucleated giant cells (blue circle). This is demonstrated on a hematoxylin and eosin stain at a magnification of 200x.

Benign epithelium and periductal condensation of atypical stromal cells was also present (Figure 6).

**Figure 7:**
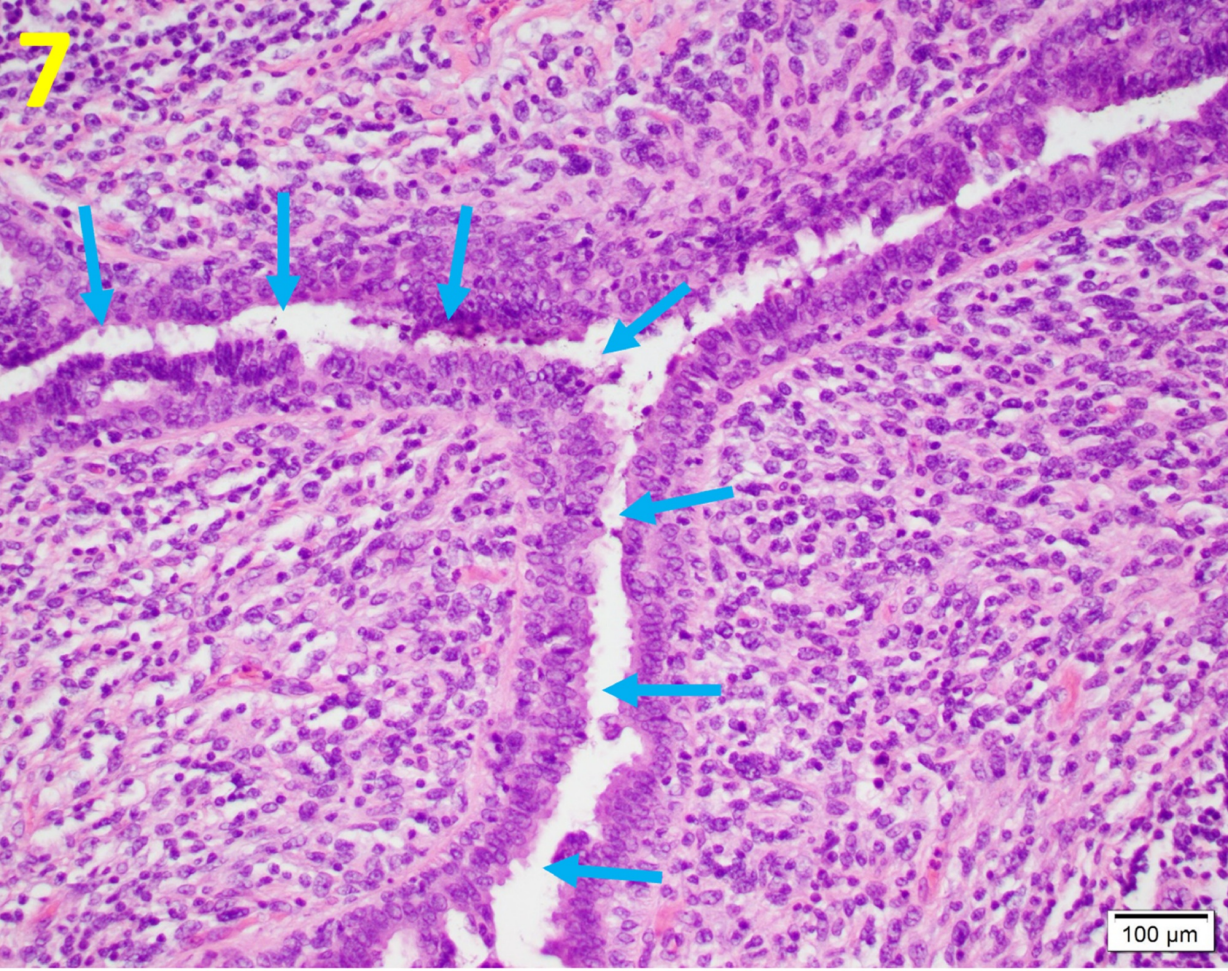
Leaf-like Projection of Phyllodes Tumors Leaf-like projection of the epithelial lining (shape formed by the blue arrows) into cystic spaces is a classic histological finding in phyllodes tumor. This is demonstrated on a hematoxylin and eosin stain at a magnification of 100x.

The tissue showed multinucleated giant cells, which are commonly seen in malignant PTs. Additionally, a leaf-like architecture and hypercellular stroma that resembled fibroblasts and myofibroblasts were noted (Figure 7).

**Figure 8:**
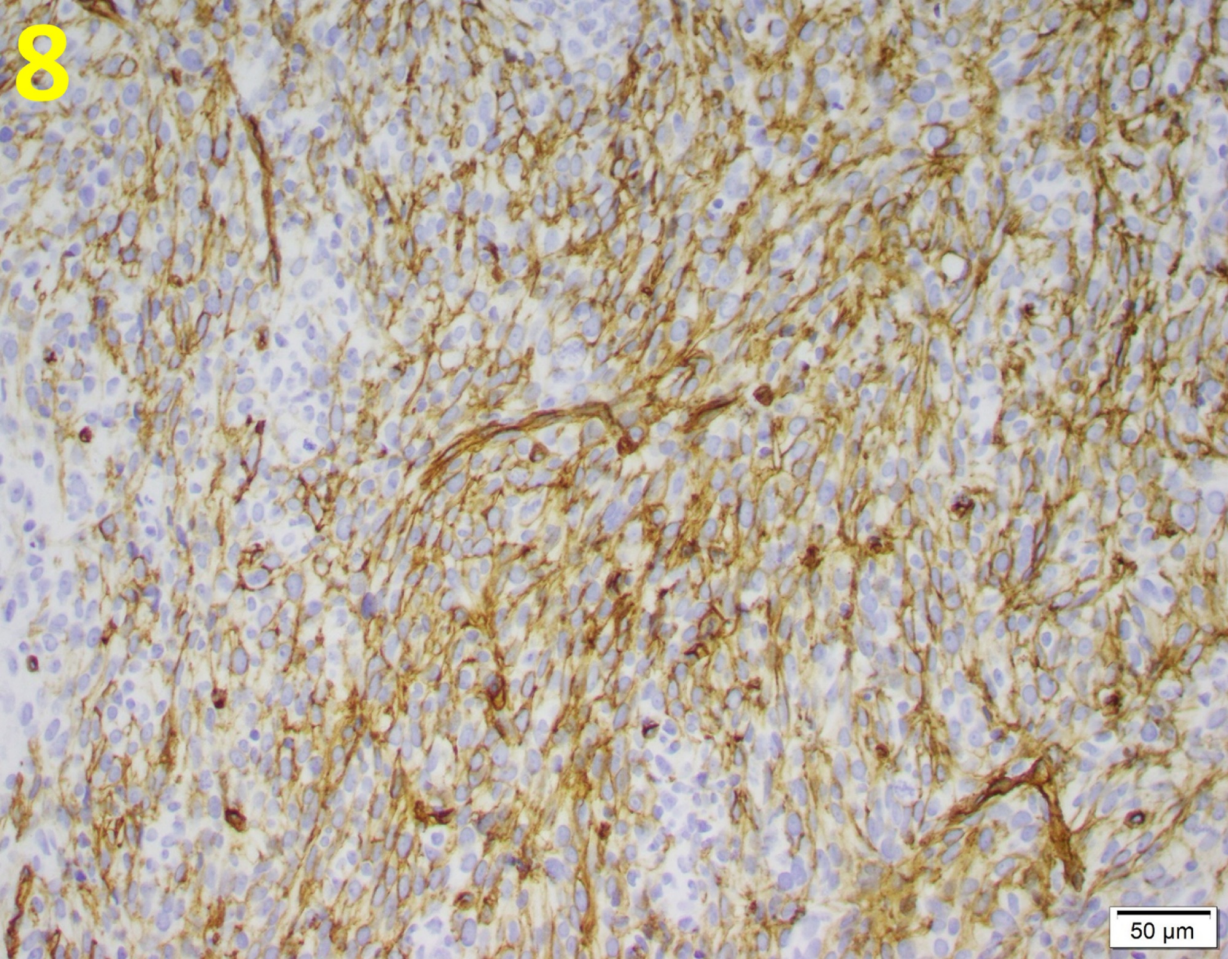
CD34 Positivity in Phyllodes Tumor CD34 positively stained spindle cells (yellow brown) are common in malignant phyllodes tumors. This is demonstrated at a magnification of 200x. CD34: cluster of differentiation 34

**Figure 9:**
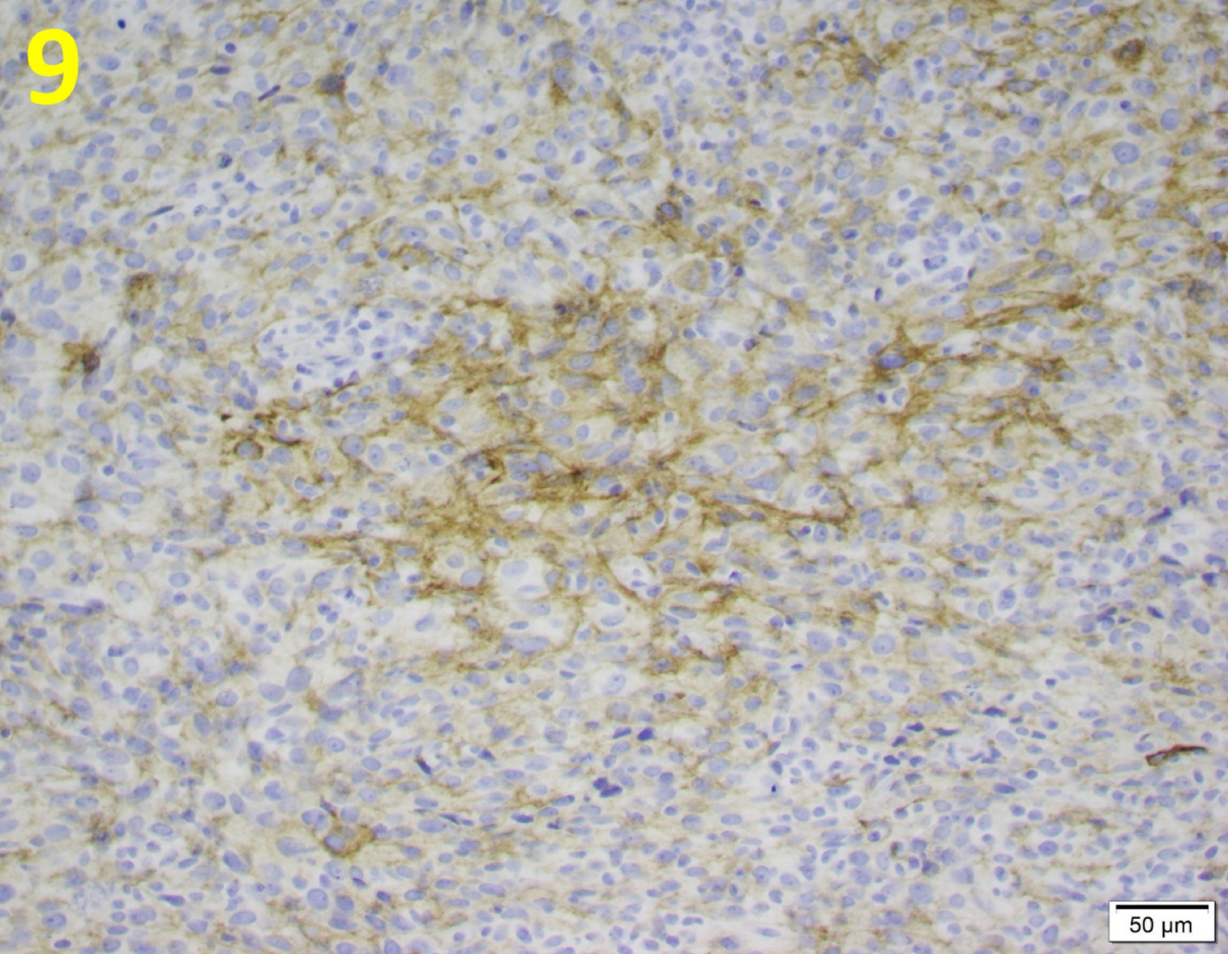
Focal Positivity for Caldesmon Stain in Phyllodes Tumor Caldesmon positivity (yellow brown) is a common histochemical finding in malignant phyllodes tumors. This is demonstrated at a magnification of 200x.

Immunohistochemical stains were diffusely positive for cluster of differentiation 34 (CD34) and vimentin and focally positive for caldesmon (Figures 8, 9).

The tissue was negative for epithelial membrane antigen, cytokeratin AE1/3, cytokeratin 7, desmin, p63 and melan-A. The morphological findings and immunohistochemical staining profile were consistent with the diagnosis of primary malignant PTs of the right breast. Beyond this, metastasis from the previously diagnosed PT from the left breast was ruled out.

The patient received one round of adjuvant radiation to the breast and was monitored for recurrence. At two years of oncologic surveillance, the patient did not show any signs of active metastasis.

## Discussion

Among the three subtypes of PT, malignant PT is the rarest entity which accounts for approximately 10% to 30% of all PTs [4]. Although malignant PT typically presents in the fourth to sixth decades of life, it can also occur in younger women. In a study completed by Abusalem et al., it was estimated that one out of 26 PTs was deemed to be malignant in patients 20-29 years of age [5,6]. Grossly, malignant PT has varying clinical presentations in size, growth pattern and changes in the overlying skin [7]. Microscopically, malignant PTs are characterized by the leaf-like protrusions and elongated cleft-like spaces that contain papillary projections of stromal overgrowth with epithelial lining [3]. Histologically, malignant PT is differentiated from other subtypes by prominent stromal overgrowth and atypia, infiltrative margins and high mitotic rate (more than 10 mitoses per 10 high-power fields) [6,8]. Other sarcomatous features including chondrosarcoma, liposarcoma and rhabdosarcoma can also be present [9]. Tumor markers for malignant PT are variable. Immunochemistry of malignant PT is often negative for p63 and cytokeratins [10]. However, studies have shown that these two markers can be positive in 57% and 21%, respectively for malignant PTs [11]. Staining positive for CD34 is another useful diagnostic tool but only seen in 37% to 57% of malignant PTs [12]. Therefore, these markers need to be carefully assessed in combination with histological features to differentiate benign from malignant tumors.

An important pathology to consider in the differential is myofibrosarcoma (MFS), which can also stain positive for CD34 and demonstrate stromal overgrowth with cellular atypia on histology. However, histopathology findings in MFS typically include medium-to-large spindle cells grouped in large and irregular fascicles, which are separated by broad strands of hyalinized collagen fibers, fat cells or myxoid matrix [13]. Additionally, the leaf-like epithelial component seen in malignant PTs is not present in MFS.

Beyond the histological components of malignant PT, the appearance of malignant PT on sonographic imaging as a smooth, poly-lobulated mass resembles a fibroadenoma (FA) [14]. More specifically, malignant masses can share common features with complex FA which present variably on ultrasound, ranging from a well-circumscribed hypoechoic mass to a heterogenous mass with features of malignancy, including ill-defined margin, lobulation, and microcalcification [14]. The presence of intralesional clefts and cystic spaces on ultrasound may favor PTs [14]. However, these characteristics have not been proven reliably useful for differentiation. On mammography, PTs are round or oval with either well- or ill-defined margins, which are very non-specific and make distinguishing PT from other neoplasms difficult. Because of the minimal values of mammography and sonography in providing an accurate diagnosis, magnetic resonance imaging (MRI) has been suggested as a useful diagnostic tool. The presence of a heterogenous inner structure and non-enhancing septations on MRI may indicate a diagnosis of PT more often than histologically proven FA [15]. Additionally, slit-like patterns in enhanced images and signal changes from T2-weighted to enhanced images are common features in PT. Breast imaging-reporting and data system (BI-RADS) categories are also strongly associated with histological grade of PTs. BI-RADS category 4a on mammography, category 3 on ultrasound and category 4b on MRI are most commonly seen in high-grade PTs [14]. BI-RADS category ≥4a is considered to be suspicious for malignancy and, hence, warrants further intervention [14].

The aforementioned features are critical for radiologists to be aware of, as FA is the most common breast tumor in adolescent and young females. Therefore, malignant PTs may oftentimes be overlooked as a potential differential diagnosis. Histologically, FA share the fibroblastic and stromal overgrowth features with malignant PT, but they have rare mitotic figures in both glandular and stromal components, which are common in malignant PT. Furthermore, stromal overgrowth in FA is generally more uniform and without cellular atypia. Malignant PTs may also have clusters of multinucleated giant cells, which are typically not present in FA.

Although not required for diagnosis, malignant PTs have been shown to be highly associated with mutations in mediator complex subunit 12 in addition to several oncogenes and tumor suppressors, including p53, retinoblastoma, neurofibromin 1 and epidermal growth factor receptor [16,17]. Cytogenic studies have also demonstrated a strong correlation between borderline/malignant PT and 1q gain, and 9p21 deletion associated with loss of p16-Ink4a expression [17,18]. This patient’s past medical history of multiple breast tumors, including a sarcoma and a borderline PT at a very young age, raises suspicion for a genetic component in the development of this rare malignant PT. Specifically, a past medical history of breast sarcoma at age 19 years is concerning for Li-Fraumeni syndrome, which is a rare genetic condition caused by p53 mutations. Li-Fraumeni syndrome has also been associated with an increased risk of developing PT [19].

The management of most cases of malignant PTs recommended by National Comprehensive Cancer Network guidelines includes complete surgical resection with negative margins of at least 1 cm. Mastectomy is generally not recommended because of concern for poor cosmetic outcomes, unless positive margins cannot be achieved or if the tumor is too large to be completely excised. Adjuvant radiation therapy is controversial in terms of preventing tumor recurrence. Radiation was found to prevent recurrence of borderline and malignant PTs following surgical interventions (breast-conserving surgery and mastectomy) but was found to have no impact on disease-free survival [20]. Because our patient had a strong family history of breast cancer and also a past medical history of multiple high-grade breast tumors, it was determined that a partial mastectomy of the right breast would be the appropriate course of treatment, followed by adjuvant radiation therapy and annual surveillance mammography.

## Conclusions

Although most PTs are benign, early detection of the malignant subtype of PT is important. A past medical history of multiple breast tumors and a significant family history raise suspicion for a genetic component in this rare entity. Current literature suggests chromosome imbalances and gene deletions as possible causes of malignant PT, but the exact etiology remains unknown. Future research focused on the hereditary aspect of malignant PT can shed light on the etiopathogenesis of this rare type of tumor and its possible relationship with other disease processes.
